# Mosaic embryos result in equivalent live birth rates when compared to euploid embryos following frozen embryo transfer

**DOI:** 10.1007/s10815-026-03808-2

**Published:** 2026-01-29

**Authors:** Shmuel Sashitzky, Sarah C. Rubin, Lauren Tetelbaun, Carolyn Robb, Rachel Stern, Moses Bibi, Victoria Rodriguez, Alexis Greene, Martin Keltz

**Affiliations:** 1 Department of Obstetrics and Gynecology, New York Medical College School of Medicine, 40 Sunshine Cottage Road, Valhalla, 10595 USA; 2 WestMed - Summit Health, Reproductive Endocrinology, Purchase, NY USA

**Keywords:** Mosaic, Euploid, IVF, Live birth, FET

## Abstract

**Purpose:**

To compare live birth rates (LBRs) between mosaic and euploid embryos.

**Methods:**

Retrospective cohort study analyzing frozen mosaic (56) and euploid (819) embryos tested with next generation sequencing, transferred between October 2018 and December 2023. The primary outcome was LBR per embryo transferred. Secondary outcomes included LBR per embryo transfer cycle, implantation rate (IR), miscarriage rate (MR), double embryo transfer (DET) rate, twin rate, high-level (HL) versus low-level (LL) mosaicism, segmental or whole chromosomal mosaicism, freeze day and grade, and neonatal outcomes. Chi-squared and student *t*-test were applied, with significance set at *p* < 0.01.

**Results:**

Per embryo, mosaic and euploid embryos had similar LBR (50.0% versus 51.8%, *p* = 0.80) and IR (55% versus 56%, *p* = 0.88). Per cycle, biochemical pregnancy (22.0% versus 17.8%, *p* = 0.41), clinical pregnancy rate (53.1% versus 56.2%, *p* = 0.77), and MR (7.7% versus 7.6%, *p* = 1.00) were not significantly different. LBR in LL versus HL mosaics was 59.4% versus 37.5% (*p* = 0.18) and 48% versus 50% for segmental versus whole chromosomal defects (*p* = 1.00). Mosaic embryos were transferred in significantly older patients (37.5 vs 36.1 years, *p* = 0.01), but age did not affect LBR after adjustment at the time of embryo transfer (*p* = 0.65). DET was more frequent with mosaic than euploid embryos (41% versus 4.8%, *p* < 0.001), yielding a higher twin LBR (21% versus 2.7%, *p* < 0.001).

**Conclusion:**

Mosaic embryos had nearly identical LBR and MR to euploid embryos, supporting transfer before repeating IVF retrieval. Given the elevated twin risk with mosaic DET, single embryo transfer should be prioritized for all tested embryos.

## Introduction

While fluorescent in situ hybridization was introduced in the 1990 s to screen for up to five chromosomes in a single cell biopsy [1, 2], preimplantation genetic screening for aneuploidy has been integrated into IVF protocols over the last 15 years. The first test enabling comprehensive chromosome screening, array comparative genomic hybridization (aCGH), became widely available about 10–15 years ago. However, aCGH was eventually replaced by next-generation sequencing (NGS) within the past 5–10 years due to its improved cost efficiency and enhanced resolution [3]. Despite advancements in technology, the goal of PGT-A remains the same: to minimize miscarriage rates (MR) and maximize live birth rates (LBR) per single embryo transfer (SET).

With NGS, results indicate partial and whole chromosome gains or losses, leading to diagnoses that report as euploid when the analysis suggests an 80–100% likelihood of a normal chromosome complement. Mosaicism, in the laboratory utilized in this study, is reported as either low-level (LL) (20–40%) or high-level (HL) (> 40–80%) mosaicism. Additionally, chromosomal defect is reported as segmental or whole. Mosaicism presents a clinical challenge, with roughly 10–30% of embryos reported as mosaic [4]. Evidence suggests that many embryos with trophectoderm biopsies classified as mosaic may in fact be euploid, as their inner cell mass, the trophectoderm, or placenta often becomes euploid when assessed at later stages of development [5]. One randomized controlled trial demonstrated that 65% of segmental mosaic embryos and 35% of whole chromosome mosaic embryos were reclassified as euploid upon rebiopsy [6]. In 2020, Lin et al. found comparable IRs and LBRs between LL mosaics (20–50% aneuploidy) and HL mosaics (50–80% aneuploidy) (44.5% versus 36%; *p* = 0.45) but reported a significantly higher MR in HL mosaics in comparison to LL mosaics (5.1% versus 30.7%; *p* = 0.012) [7]. In 2021, a prospective trial by Capalbo et al. found non-inferior outcomes for MET (*N* = 413) in comparison to EET (*N* = 484) when assessing LBR, pregnancy loss, and chromosomal abnormalities in pregnancy and in children. They reported LBRs as 43.3% for euploid embryos, 42.9% for LL mosaics (20–30% aneuploid cells), and 42% for ML mosaics (30–50% aneuploid cells) [8]. Also in 2021, Viotti et al. found more favorable outcomes with segmental mosaic embryos than whole chromosome mosaic embryos, but with no significant differences (IR: 51.6% versus 41.8%, ongoing pregnancy/birth: 43.1% versus 31.3%) [4]. In 2025, Shen et al. reported that LL mosaic embryos (20–49%) have similar clinical pregnancy and live birth rates compared to euploid embryos [9], and Geng et al. reported that high morphological scoring mosaic embryos (≥ 4BB, per Gardner’s scoring system) have similar clinical and biochemical pregnancy and live birth rates compared to low scoring euploid embryos (4BC) [10].

In clinical practice, decisions regarding mosaic embryo transfer often involve shared decision-making between clinicians and patients, particularly when euploid embryos are unavailable. For many patients, the potential for a live birth outweighs the risks of lower live birth rates (LBRs) or higher miscarriage rates (MRs). While there is data currently to suggest mosaic embryo transfer outcomes are comparable to euploid embryos, the research is still limited, and guidance regarding high-level and low-level mosaics and live birth-related outcomes is underexplored. In our practice, we have been using mosaic embryos with non-viable aneuploidic mosaicism for the past 5 years when no euploid embryos were available for transfer, or when a patient requested DET of a euploid and a mosaic. Anecdotally, we did not notice poor outcomes with mosaic embryo transfer. This project adds novelty to the current literature by filling gaps in providing outcomes pertaining to high-level and low-level mosaics and live births when comparing mosaic and euploid embryo transfers. We hypothesize that mosaic embryo transfer will be associated with similar live birth rates when compared to euploid embryo transfer.

## Materials and methods

We conducted a single-institution retrospective cohort study. All patients who underwent a frozen embryo transfer (FET) of at least one mosaic embryo between October 2018 and December 2023 were compared to all patients who underwent an FET of at least one euploid embryo in the same time frame.

Approval from the Institutional Review Board (IRB) was obtained prior to the initiation of the study. All patients who met inclusion criteria between October 2018 and December 2023 were analyzed. Inclusion criteria were all women aged 18 to 42 years old who underwent an in vitro fertilization (IVF) cycle resulting in a euploid or mosaic embryo confirmed by preimplantation genetic testing for aneuploidy (PGT-A) and with a subsequent frozen embryo transfer (FET). All biopsies and embryo freezing were performed at the same laboratory, and all PGT-A testing was conducted through CooperGenomics, using consistent techniques throughout the study period.

In the menstrual cycle prior to IVF, patients received oral contraceptive pills (OCPs) in preparation for either a Gonadotropin-Releasing Hormone (GnRH) Antagonist or a GnRH Agonist Flare protocol. Protocol and dosing of gonadotropins were based on doctor preference and patient’s prior IVF cycles.

Controlled ovarian hyperstimulation (COH) was initiated with exogenous gonadotropins. The dose of gonadotropins, recombinant FSH (rFSH) and/or human menopausal gonadotropins (HMG), was individually adjusted based on ovarian response. Transvaginal sonography and serial estradiol (E2) levels were used to monitor ovarian follicular development. Once a dominant follicle reached a diameter of 18 to 20 mm, 2500–10,000 IU of hCG was administered based upon the patient’s individual weight and risk for OHSS. Transvaginal ultrasound-guided oocyte retrieval was performed 36 h later. Insemination was performed with either standard insemination or intracytoplasmic sperm injection (ICSI) based on male factor indications. Fertilization was confirmed 20 h later by the presence of two pronuclei (2PN). Embryos were kept in culture until day 3–4, when they underwent laser-assisted hatching. Day 5–7 blastocysts underwent trophectoderm biopsy and were subsequently frozen. Biopsy samples were sent to CooperGenomics (CooperSurgical Fertility Solutions, Livingston, NJ) for PGT-A testing, which reported back NGS data.

Embryo transfers were performed in a subsequent FET cycle. Estradiol at increasing doses up to 4 mg BID over a 2-week period was used to thicken the endometrial lining until it appeared both triple-layered and thickened to ideally 8–10 mm. Leuprolide acetate 14-day kits were used in about half the cases in order to further thin the lining prior to estradiol start and prevent ovulation when patients were known to have endometriosis or irregularly thickened endometrial linings. Luteal phase supplementation with IM or vaginal progesterone was given to all patients starting at 50 mg of progesterone in oil daily starting in the evening 5 days prior to embryo transfer. Serum β-hCG levels were tested on days 9–12 after embryo transfer. If serum β-hCG was positive, transvaginal sonography was performed on day 19 to confirm the presence of the gestational sac. Only pregnancies that resulted in sonographically confirmed gestational sacs were considered clinical pregnancies; biochemical pregnancies were counted as an IVF failure. Ongoing pregnancies were those that continued on with a normal fetal heart rate into the second trimester. Live births were confirmed with the vaginal delivery or cesarean section operative report.

Pregnancy outcomes of each cycle were obtained, including clinical pregnancies, defined as the presence of a gestational sac on the ultrasound, ongoing pregnancies, defined as a viable fetus in the second trimester, and the number of miscarriages, defined as pregnancy loss prior to 20 gestational weeks. Implantation rate was calculated by dividing the number of embryos resulting in a confirmed implantation—defined as a clinical intrauterine, ectopic, or heterotopic gestation with at least one fetal heartbeat—by the number of embryos transferred. Cycles were excluded from both the numerator and denominator if it was impossible to attribute implantation to a specific embryo type with 100% certainty. Ambiguous cycles were included only when implantation could be confidently assigned based on fetal count and manual chart review (e.g., fetal sex). The primary outcome was LBR per embryo transferred. Secondary outcomes included LBR per embryo transfer cycle, miscarriage rate (MR), twin live birth rate (LBR), double embryo transfer (DET) rate, and neonatal outcomes including birth weight and congenital anomalies. Additionally, embryos were analyzed by high-level (HL) versus low-level (LL) mosaicism, segmental or whole chromosomal mosaicism, freeze date, and grade. Embryos were graded using the modified Gardner ABCD blastocyst grading system, which evaluates blastocyst expansion, inner cell mass (ICM) quality, and trophectoderm (TE) quality [11]. Under this system, embryos with A or B grades in both the ICM and TE compartments and no C components were classified as “good” quality (e.g., A/A, A/B, B/A, B/B). Embryos with one B and one C grade (B/C or C/B) were categorized as “fair” quality, while those with C/C grades for both the ICM and TE were classified as “poor” quality. For analysis in Table 2, embryo quality was grouped into “good” versus “fair/poor” categories based on these criteria.

All data were analyzed using RStudio programming software. Statistics employed chi-squared for categorical and student *t*-test for continuous data. Statistical significance in our primary outcome used *p* ≤ 0.05, while our secondary outcomes required *p* ≤ 0.01 due to multiple significance testing. Power analysis confirmed that, with 819 euploid and 56 mosaic embryos (14:1 ratio), the study was adequately powered to exclude an absolute reduction in live birth rate of 20%, given a 50% rate for euploids and 30% for mosaics. To evaluate whether patient age influenced pregnancy outcomes between euploid and mosaic embryo transfers, we used RStudio to construct a generalized linear model with euploid vs mosaic embryo group as the primary predictor and patient age at time of embryo transfer as a covariate.

## Results

A total of 56 mosaic embryos and 819 euploid embryos were transferred in 49 and 793 transfer cycles, respectively. Patients who underwent mosaic versus euploid embryo transfer were similar in baseline characteristics (Table 1). The average age at the time of oocyte retrieval was 36.3 ± 3.7 years in the mosaic group and 35.3 ± 3.6 years in the euploid group (*p* = 0.05). Likewise, the average age at embryo transfer was slightly higher in the mosaic group (37.5 ± 3.4 years) compared with the euploid group (36.1 ± 3.7 years, *p* = 0.01). Body mass index did not differ between groups (28.3 ± 5.2 vs 27.9 ± 6.3, *p* = 0.72), while gravidity was modestly higher among patients receiving mosaic embryos (1.9 ± 1.4 vs 1.4 ± 1.4, *p* = 0.02). Racial and ethnic distributions were comparable across groups, with no statistically significant differences in any SART racial category (Table 1).

**Table 1:** Baseline characteristics per frozen embryo transfer cycle: mosaic vs euploid

Characteristic	Mosaic (*N* = 49)	Euploid (*N* = 793)	*p*-value
**Continuous variables (mean ± SD)**			
Age at retrieval	36.3 ± 3.7	35.3 ± 3.6	0.05*
Age at embryo transfer	37.5 ± 3.4	36.1 ± 3.7	0.01*
Body mass index	28.3 ± 5.2	27.9 ± 6.3	0.72
Gravidity	1.9 ± 1.4	1.4 ± 1.4	0.02*
**Categorical variable: SART racial categories (% (*n*/*N*))**			
American Indian/Alaska Native	0% (0/49)	0.4% (3/793)	1.00
Asian	6.1% (3/49)	7.1% (56/793)	1.00
Black/African American	6.1% (3/49)	7.4% (59/793)	1.00
Hispanic/Latino	16.3% (8/49)	13.7% (109/793)	0.61
Native Hawaiian/Other Pacific Islander	0% (0/49)	0.1% (1/793)	1.00
White	55.1% (27/49)	46.8% (371/793)	0.26
Unknown	12.2% (6/49)	17.4% (138/793)	0.35

*Indicates statistical significance *p* < 0.05

Because mosaic transfers occurred in slightly older patients, we conducted a generalized linear model adjusting for patient age at the time of embryo transfer. In this model, embryo type (euploid vs mosaic) was not a significant predictor of pregnancy outcomes (*p* = 0.65), indicating that the observed age differences did not confound the relationship between embryo status and reproductive outcomes.

When comparing the mosaic embryo and euploid embryo transfer groups, there was no statistically significant difference in implantation rates (56% vs 55%, *p* = 0.99), as shown in Table 2. Similarly, there was no statistically significant difference in biochemical pregnancy, clinical pregnancy, or miscarriage rates between the mosaic and euploid embryo groups (Table 2). Live birth rate was calculated per cycle and per embryo; there were no statistically significant differences in live birth outcomes between the mosaic and euploid embryo groups (Table 2). The mosaic embryo group had a higher rate of DET when compared to the euploid group (41.0% vs 4.8%, *p* < 0.001). Furthermore, mosaic embryos had an increased twin live birth rate when compared to euploid embryos (21.0% vs 2.7%, *p* < 0.001). Of note, the number of clinical intrauterine gestations is less than live births, which is likely due to multiple pregnancies.

**Table 2:** Comparison of outcomes of mosaic and euploid embryo transfer

	Mosaic embryos	Euploid embryos	*p*-value
Live birth rate per embryo transferred	50.9% (28/55)	51.8% (424/818)	0.80
Live birth rate per cycle	49.0% (24/49)	52.0% (412/793)	0.69
Implantation rate per embryo transferred	55.0% (30/55)	56.0% (455/818)	0.88
Biochemical pregnancy rate per cycle	22.0% (11/49)	17.8% (141/793)	0.41
Clinical pregnancy rate per cycle	53.1% (26/49)	56.2% (446/793)	0.77
Miscarriage rate per cycle	7.7% (2/26)	7.6% (34/446)	1.00
Double embryo transfer rate per cycle	41.0% (20/49)	4.8% (38/793)	< 0.0001*
Twin rate per live birth	21.0% (5/24)	2.7% (11/412)	< 0.001*

*Indicates statistical significance *p* < 0.05

In Table 2, the denominator for live birth rate per embryo transferred represents the total number of embryos transferred (mosaic, *n* = 55; euploid, *n* = 818). For live birth rate per cycle, biochemical pregnancy rate, clinical pregnancy rate, and double embryo transfer rate, the denominator represents the total number of embryo transfer cycles (mosaic, *n* = 49; euploid, *n* = 793). Implantation rate is calculated per embryo transferred resulting in implantation, with denominators reflecting the number of embryos transferred (mosaic, *n* = 55; euploid, *n* = 818). Miscarriage rate is calculated per clinical pregnancy, with denominators representing the number of clinical pregnancies (mosaic, *n* = 26; euploid, *n* = 446). Twin rate is calculated per live birth, with denominators representing the number of live birth cycles (mosaic, *n* = 24; euploid, *n* = 412).

When comparing outcomes by the day of embryo freeze, mosaic embryos frozen on days 6 or 7 resulted in a lower, though not statistically different, live birth rate compared to embryos frozen on day 5 (52.2% vs 48.5%, *p* = 1.0), as shown in Table 3. When stratifying by degree of mosaicism, mosaic embryos with low-level defects had a higher, though not statistically different, live birth rate than those with high-level defects (59.4% vs 37.5%, *p* = 0.11). Mosaic embryos with whole chromosome (50.0%) or segmental (48.4%) abnormalities did not differ significantly in live birth rate (*p* = 0.18). Embryo grade also showed a non-significant trend, with “good” embryos achieving a higher live birth rate than “fair and poor” embryos (57.1 vs 38.1%, *p* = 0.27).

**Table 3:** Live birth outcomes for day of transfer, chromosomal defect, mosaicism level, and embryo grade among mosaic embryo transfers

**Day of freeze**			
	Day 5	Day 6/7	*p*-value
Live birth rate	52.2% (12/23)	48.5% (16/33)	1.0
**Chromosomal defect**			
	Whole	Segmental	*p*-value
Live birth rate	50.0% (11/22)	48.4% (15/31)	1.0
**Mosaicism level**			
	High level	Low level	*p*-value
Live birth rate	37.5% (9/24)	59.4% (19/32)	0.18
**Embryo grade**			
	Good	Fair and poor	*p*-value
Live birth rate	57.1% (20/35)	38.1% (8/21)	0.27

A total of 56 mosaic embryos were transferred in the study; in Table 3, denominators in each category reflect the number of embryos for which complete classification data were available. For the comparison of day of freeze, all 56 embryos were included (23 day-5 embryos and 33 day-6/7 embryos). For the analysis of chromosomal defect, embryos were categorized as either whole chromosome or segmental defects; three embryos were classified as mixed mosaicism and therefore excluded from this comparison, resulting in 22 whole chromosome and 31 segmental embryos. Mosaicism level analysis includes all embryos with available high-level or low-level mosaic classifications (24 high-level and 32 low-level embryos). Embryo grade analysis includes embryos assigned to good versus fair/poor morphological categories (35 good-grade and 21 fair/poor-grade embryos).

Neonatal outcomes were comparable between mosaic and euploid embryo transfers. Mean birth weight did not differ significantly between groups, with infants born from mosaic embryos weighing 3194.7 ± 707.8 g and those from euploid embryos weighing 3376.1 ± 595.1 g (*p* = 0.19). Importantly, no congenital anomalies, including genetic defects, cleft palate, limb defects, cardiac defects, or neural tube defects, were reported in either group during the study period.

## Discussion

This study demonstrates that mosaic embryos achieve live birth rates (LBR) comparable to euploid embryos following frozen embryo transfer (FET), with nearly identical LBRs of 50% and 51.8%, respectively (*p* = 0.91). Similarly, implantation rates, biochemical pregnancy rates, and clinical pregnancy rates showed no statistically significant differences between the two groups, further supporting the viability of mosaic embryos. Notably, our findings suggest that the level of mosaicism—whether low level or high level—did not result in significant differences in LBR, although there was a trend favoring LL mosaicism (*p* = 0.18). Additionally, outcomes such as miscarriage rates and live birth outcomes were consistent across groups, despite mosaic embryos being transferred in older patients. Further, our results showed age at embryo transfer was significantly greater than age at embryo retrieval for patients in the mosaics group. This increase in age can be attributed to patients attempting transfer or giving birth with euploid oocytes prior to mosaic transfer. Importantly, the higher twin rates observed in mosaic transfers are largely due to the increased use of DETs, often involving two mosaic embryos in an effort to increase the likelihood of pregnancy rather than intrinsic biological properties of mosaic embryos. This underscores the recommendation that SET should be prioritized, even for mosaic embryos. These results reinforce the clinical viability of transferring mosaic embryos and highlight the potential to avoid repeated IVF cycles by utilizing available mosaic embryos, thereby reducing patient burden and optimizing outcomes.

To understand why mosaic embryos can result in healthy live births, researchers have proposed several hypotheses. One theory suggests that embryos possess the ability to self-correct, with euploid cells outcompeting aneuploid ones through mechanisms like apoptosis and autophagy [12]. However, another explanation points to limitations in the testing process itself. NGS determines mosaicism by inferring intermediate chromosomal copy numbers, but factors such as test artifacts, amplification bias, contamination, and variability in laboratory techniques can produce false positives. Moreover, the cells sampled from the trophectoderm may not accurately represent the genetic composition of the inner cell mass, which ultimately forms the fetus [13]. These limitations highlight the need for careful interpretation of NGS results. A study by Girardi et al. found that a 50% threshold offered the most accuracy, eliminating the mosaic result entirely in favor of binary euploid or aneuploid outcomes [14]. These findings suggest that refining thresholds could improve the clinical utility of NGS and reduce confusion regarding mosaicism classification.

Earlier studies have shown that euploid embryos have higher implantation and live birth rates [15] and lower miscarriage rates than mosaic embryos [16]; however, in 2025, Shen et al. reported that LL mosaic embryos (20–49%) have similar clinical pregnancy and live birth rates compared to euploid embryos [9], and Geng et al. reported that high morphological scoring mosaic embryos (≥ 4BB, per Gardner’s scoring system) have similar clinical and biochemical pregnancy and live birth rates compared to low scoring euploid embryos (4BC) [10]. While our study evaluated mosaic embryos as a single combined group rather than separating low- and high-level embryos, we found that overall mosaic outcomes were comparable to those of euploid embryos. Furthermore, previous research reports findings similar to our results showing comparable outcomes for mosaic embryo transfer (MET) and euploid embryo transfer (EET). A prospective trial by Capalbo et al. [8] found non-inferior outcomes for MET (*N* = 413) in comparison to EET (*N* = 484) when assessing LBR, pregnancy loss, and chromosomal abnormalities in pregnancy and in children. They reported LBRs as 43.3% for euploid embryos, 42.9% for LL mosaics (20–30% aneuploid cells), and 42% for ML mosaics (30–50% aneuploid cells), which is comparable to our findings. In addition, a retrospective study by Lin et al. found comparable IRs and LBRs between LL mosaics (20–50% aneuploidy) and HL mosaics (50–80% aneuploidy) (44.5% versus 36%; *p* = 0.45) but reported a significantly higher MR in HL mosaics in comparison to LL mosaics (5.1% versus 30.7%; *p* = 0.012) [7]. One difference is that their HL mosaics were 50–80% aneuploidy whereas our HL mosaics were > 40–80% aneuploidy, which may account for the discrepancies in MR. Our findings are additionally consistent with Viotti et al., who found more favorable outcomes with segmental mosaic embryos than whole chromosome mosaic embryos, but with no significant differences (IR: 51.6% versus 41.8%, ongoing pregnancy/birth: 43.1% versus 31.3%) [4]. Finally, our data showed a higher rate of DETs with mosaic embryos than euploid embryos, likely due to prior literature advising against DETs with euploid embryos due to increased twin pregnancy rates [17]. Previous studies have investigated euploid SETs versus DETs [17]; however, there is a lack of research investigating mosaic DETs versus mosaic SETs. As our findings demonstrate favorable outcomes for METs, mosaic SETs should be prioritized over mosaic DETs to prevent the risk of multiple gestations.

Neonatal outcomes in our cohort were reassuring and consistent with the broader literature demonstrating the safety of transferring mosaic embryos. We observed no significant difference in birth weight between infants born after mosaic versus euploid embryo transfer, aligning with multiple multicenter analyses and committee opinions showing no differences in average birth weight or gestational length between the two groups [18, 19]. These findings echo the American Society for Reproductive Medicine’s guidance that enhanced fetal growth restriction surveillance after mosaic embryo transfer is not warranted based on current evidence [20]. Importantly, no congenital anomalies, including cardiac, limb, neural tube, or genetic defects, were identified in either group, consistent with large cohort studies and systematic reviews reporting very low and comparable rates of congenital anomalies following mosaic and euploid embryo transfer [18–20]. In one of the largest multicenter studies, only one gross abnormality was observed among 488 infants born after mosaic embryo transfer, underscoring the rarity of adverse structural outcomes [18]. Overall, both our findings and the existing literature support that mosaic embryo transfer does not appear to increase the risk of adverse neonatal outcomes or congenital anomalies compared with euploid embryo transfer.

This study has notable strengths. First, conducting the study at a single institution ensures consistency in the procedures performed and makes it easier to track and monitor data. Second, the study’s retrospective design prevented the risk of losing patients to follow-up and therefore included outcomes for all of the included patients. Our study also had a few limitations: although having data from a single center ensures consistency, it may also limit generalizability to other IVF centers due to factors such as patient demographics. The inclusion of one center additionally limited the number of mosaic embryos, which could have reduced the study’s power to detect statistically significant differences between euploid and mosaic embryos. However, we performed a power analysis, which confirmed that, with 819 euploid and 56 mosaic embryos (14:1 ratio), the study was adequately powered to exclude an absolute reduction in live birth rate of 20%, given a 50% rate for euploids and 30% for mosaics. In addition, the retrospective nature of our study may have not accounted for patient selection bias, such as MET when no euploid embryos were available, or MET in patients of advanced maternal age. However, a generalized linear model adjusting for patient age at the time of embryo transfer showed that embryo type (euploid vs mosaic) was not a significant predictor of pregnancy outcomes (*p* = 0.65), indicating that the observed age differences did not confound the relationship between embryo chromosomal status and reproductive outcomes. Additionally, we included all mosaic and euploid embryos from the study period, reducing selection bias. To address the limitations of our study, prospective randomized trials with large sample sizes are needed to assess clinical outcomes for the transfer of mosaic versus euploid embryos. Furthermore, we hope to conduct larger studies in the future, incorporating the day of freeze and embryo grade for euploid and mosaic embryos, and high-level vs low-level and whole vs partial chromosomal defects for mosaic embryos to create an algorithm to determine the optimal embryo to transfer.

This study demonstrates that mosaic embryos can achieve comparable live birth rates to euploid embryos following frozen embryo transfer. Our findings suggest that the level of mosaicism (low or high) and the type of mosaicism (segmental or whole chromosome) do not significantly impact live birth rates. Importantly, the higher twin rates observed with mosaic embryos emphasize the need to prioritize single embryo transfers, even in this population. These results support the clinical viability of transferring mosaic embryos, particularly when euploid embryos are unavailable, to avoid repeated IVF cycles and reduce patient burden. Based on our data and the growing body of literature supporting mosaic embryo transfers, clinicians should confidently counsel patients on the potential for success with these embryos and prioritize their use to optimize IVF outcomes. Given the equivalent live birth rate found between mosaic and euploid embryos, we suggest that mosaic embryos should be considered for transfer prior to repeating IVF retrieval for euploids, which can minimize costs as well as the physical and emotional impact on patients. Additionally, the high rate of twin deliveries observed with mosaic double embryo transfers suggests single embryo transfer should be utilized, which would minimize risks associated with multiple gestations.

## Data Availability

Data sets are fully accessible on SART and CooperGenomics.
